# Supplementary material for: Immunosurveillance and molecular detection of hepatitis B virus infection amongst vaccinated children in the West Gonja District in Savanna Region of Ghana
Source: PLoS One. 2021 Sep 17;16(9):e0257103. doi: 10.1371/journal.pone.0257103 (PMC8448355; doi:10.1371/journal.pone.0257103)
Supplement: S1 File — (PDF) [file pone.0257103.s001.pdf]

QUESTIONNAIRE

KWAME NKRUMAH UNIVERSITY OF SCIENCE AND TECHNOLOGY

SCHOOL OF MEDICINE AND DENTISTRY

DEPARTMENT OF CLINICAL MICROBIOLOGY

Please read before completing the questionnaire

This is a questionnaire designed to find out the effectiveness of hepatitis B vaccination programme in West Gonja District of Ghana. This would help provide information for a project to determine immune response and duration of protection of hepatitis B vaccination. Please know that any information given will be kept confidential and use only for academic purposes.

NB: The questionnaire should be completed by research participants aged nine (9) months to seventeen (17) years with assistance from parents or guardians.

Date: DD………/MM………. /YY……………….
Code…………………………………………………..

Please check (√) only the box that most correctly answers the question making sure you make only one selection from each question except otherwise indicated

SECTION A

Personal information

1. Gender: Male ☐ Female ☐
2. Age ………………………………………….
3. Residential area …………………………………

SECTION B

Hepatitis B virus immunity in relation to a child's medical history

4. Hospital admission Yes ☐ No ☐
5. Open abscess Yes ☐ No ☐
6. Surgical operation Yes ☐ No ☐
7. Diabetic patient Yes ☐ No ☐
8. Renal failure Yes ☐ No ☐
9. Cancer Yes ☐ No ☐
10. Hemodialysis patient Yes ☐ No ☐
11. Did the child have a blood transfusion? Yes ☐ No ☐
12. Retroviral Screen Positive ☐ Negative ☐ Not done ☐
13. Hepatitis B status of mother Positive ☐ Negative ☐

SECTION C

14. Is this the first time the child has been tested for hepatitis B virus? Yes ☐ No ☐

15. If no, when was the client last tested for hepatitis B?
Date…………………………………

16. Have you ever heard of hepatitis B vaccination? Yes ☐ No ☐

17. How effective do you think hepatitis B vaccination is in protecting someone against hepatitis B virus infection?

- ☐ Not effective
- ☐ Strongly effective
- ☐ Slightly effective
- ☐ Very effective
- ☐ I don't know

18.Have you ever received hepatitis B vaccination? Yes ☐ No ☐

19.If your answer to question 18 is yes, how many doses of hepatitis B vaccination have you received.

- ☐ One dose
- ☐ Two doses
- ☐ Three doses
- ☐ More than three doses

20.When did you receive the last dose of hepatitis B vaccination?

- ☐ Less than one month ago
- ☐ One month to three months ago
- ☐ Four month to six months ago
- ☐ More than six months ago

21.How long does a full dose of hepatitis B vaccine give protection?

- ☐ Less than one year
- ☐ One year to five years
- ☐ Six to ten years
- ☐ Eleven to nineteen years
- ☐ Twenty years or more
- ☐ I don't know

Thank you for your time.!!!
