# Supplementary material for: Immunosurveillance and molecular detection of hepatitis B virus infection amongst vaccinated children in the West Gonja District in Savanna Region of Ghana
Source: PLoS One. 2021 Sep 17;16(9):e0257103. doi: 10.1371/journal.pone.0257103 (PMC8448355; doi:10.1371/journal.pone.0257103)
Supplement: Attachment — Submitted filename: RESPONSE TO REVIEWER(S).docx [file pone.0257103.s002.docx]

20^th^ July, 2021

The Editor in Chief

PLoS One Journal

Dear Editor in Chief,

**SUBMISSION OF REVISED MANUSCRIPT FOR PUBLICATION**

I would like to submit a revised version of our manuscript titled ‘Immunosurveillance and molecular detection of hepatitis B virus infection amongst vaccinated children in the West Gonja District in Savanna Region of Ghana by Theophilus Quaye, Patrick W. Narkwa, Seth A. Domfeh, Gloria Kattah and Mohamed Mutocheluh to be considered for publication as a research article in the PLoS One Journal.

We thank the academic editor and the reviewer(s) for their generous comments on the manuscript. We have edited the manuscript to address their concerns.

Below are the full responses to the comments of the academic editor and the reviewer(s).

**Academic editor’s comments and responses**

Comment 1

Please ensure that your manuscript meets PLOS ONE's style requirements, including those for file naming.

Response

The manuscript has been carefully checked against PLOS ONE’s style requirement and we believe these requirements have been duly met.

Comment 2

Please include additional information regarding the survey or questionnaire used in the study and ensure that you have provided sufficient details that others could replicate the analyses. For instance, if you developed a questionnaire as part of this study and it is not under a copyright more restrictive than CC-BY, please include a copy, in both the original language and English, as Supporting Information.

Response

The questionnaire that was developed and used in the study has been provided as supporting information.

Comment 3

 We note that you have included the phrase “data not shown” in your manuscript. Unfortunately, this does not meet our data sharing requirements. PLOS does not permit references to inaccessible data. We require that authors provide all relevant data within the paper, Supporting Information files, or in an acceptable, public repository. Please add a citation to support this phrase or upload the data that corresponds with these findings to a stable repository (such as Figshare or Dryad) and provide and URLs, DOIs, or accession numbers that may be used to access these data. Or, if the data are not a core part of the research being presented in your study, we ask that you remove the phrase that refers to these data.

Response

The sentence which was referenced as ‘data not shown’ has been deleted as it does not form a core part of the study.

**Review comments and responses**

Comment 1

The introduction has the overall context and approach of importance of vaccination HBV.

Response

Not Applicable

Comment 2

In the study design and study site section, the authors should include a further description of the geographical area where the study was carried out. Consider aspects of temperature, organization, size of the city, population or another information that authors considers important.

Response:

The study design and site section have accordingly been updated with the description as suggested by the reviewer in the revised manuscript.

Comment 3

In the study population and eligibility criteria to include how much is the n of the population from 9 months to 17 years and of these how many are fully vaccinated. What was the sample size calculation? Indicate the prevalence for hepatitis B infection and the power considered to calculate the sample size. Additionally, describe in detail the inclusion criteria for the study.

Response

Data on how much is the n of the population aged 9 months to 17 years was very difficult to obtain as data from Ghana Statistical Service 2010 Population and Housing Census only indicated that the population of the West Gonja District is youthful with14.6% being between the ages of 0-4 years. The prevalence of hepatitis B infection and the power considered in calculating the sample size has been indicated in the revised manuscript. Also the inclusion and exclusion criteria have been described.

Comment 4

In the laboratory analysis section to include the number of replicates of the experiment and how the authors confirmed the results.

Response
The number of replicates and how the ELISA tests results were confirmed have been indicated in the revised manuscript.

Comment 5

In the DNA detection section, which controls were used. Mention the sensitivity and specificity of the molecular test.

Response

The controls used in RT-PCR have been indicated in the revised manuscript. The sensitivity and specificity of the molecular test as indicated by the manufacturer of the kits have been stated.

Comment 6

In the results section, explain in detail how many children were initially screened and how many didn't have the inclusion criteria.

Response

The number of children who were screened and met the inclusion criteria have explained in the revised version of the manuscript.

Comment 7

It is suggested to the authors for the presentation of the results of Table 3, to make a flow chart that writes down in better detail the results obtained in the serological and molecular study. This table does not have an adequate presentation.

Response

The focus of Table 3 is to look at the distribution of serological and molecular markers of HBV in relation to the demographics of the study participants. Several options including graphical presentation as well as use of flow chart (as suggested by the reviewer) in presenting the data were considered initially. But after careful analysis, we observed that using graphs or flow chart in presenting the data would rather have some information hidden which would make it a bit difficult for readers to comprehend. We are therefore of the opinion that presenting the data in the table as has been done in Table 3 better makes it easier to understand the information that we want to put across.

Comment 8

The conclusion describes that 86% of the participants seroconverted after primary vaccination. Could the authors explain how was obtained this result?

Response

The sero-conversion rate was determined by dividing the number of participants which had anti-HBs titers > 1mIU/mL by the total number of participants that is 301 divided by 350. However since the focus of the study was on the level of protection gained after hepatitis B vaccination, the sero-conversion part in the conclusion has been removed as the sero-conversion analysis was not captured under the result section.

We believe the manuscript is now suitable for publication in PLoS One Journal.

Sincerely yours,

SIGNED

Patrick W. Narkwa (PhD)

(Corresponding author)
